# The Residual Innate Lymphoid Cells in NFIL3-Deficient Mice Support Suboptimal Maternal Adaptations to Pregnancy

**DOI:** 10.3389/fimmu.2016.00043

**Published:** 2016-02-19

**Authors:** Selma Boulenouar, Jean-Marc Doisne, Amanda Sferruzzi-Perri, Louise M. Gaynor, Jens Kieckbusch, Elisa Balmas, Hong Wa Yung, Shagayegh Javadzadeh, Léa Volmer, Delia A. Hawkes, Keli Phillips, Hugh J.M. Brady, Abigail L. Fowden, Graham J. Burton, Ashley Moffett, Francesco Colucci

**Affiliations:** ^1^Centre for Trophoblast Research, University of Cambridge, Cambridge, UK; ^2^Department of Obstetrics and Gynaecology, University of Cambridge School of Clinical Medicine, National Institute for Health Research Cambridge Biomedical Research Centre, The Rosie Hospital, Cambridge, UK; ^3^Department of Physiology, Development and Neuroscience, University of Cambridge, Cambridge, UK; ^4^University of Cambridge Metabolic Research Laboratories, Cambridge, UK; ^5^Department of Life Sciences, Imperial College London, London, UK; ^6^Department of Pathology, University of Cambridge, Cambridge, UK

**Keywords:** mouse models, uterine NK cells, placenta, pregnancy, lymphocyte subpopulations

## Abstract

Uterine NK cells are innate lymphoid cells (ILC) that populate the uterus and expand during pregnancy, regulating placental development and fetal growth in humans and mice. We have recently characterized the composition of uterine ILCs (uILCs), some of which require the transcription factor NFIL3, but the extent to which NFIL3-dependent cells support successful reproduction in mice is unknown. By mating *Nfil3*^−/−^ females with wild-type males, here we show the effects of NFIL3 deficiency in maternal cells on both the changes in uILCs during pregnancy and the downstream consequences on reproduction. Despite the presence of CD49a^+^Eomes^−^ uILC1s and the considerable expansion of residual CD49a^+^Eomes^+^ tissue-resident NK cells and uILC3s in pregnant *Nfil3*^−/−^ mice, we found incomplete remodeling of uterine arteries and decidua, placental defects, and fetal growth restriction in litters of normal size. These results show that maternal NFIL3 mediates non-redundant functions in mouse reproduction.

## Introduction

Human and murine uterine NK cells (uNK) are innate lymphoid cells (ILCs) that produce factors active on both uterine arteries and fetal placental cells (1–3), contributing to successful reproduction (4–6). The role of uNK in pregnancy may have coevolved with that of variable immune system genes in reproduction (7, 8). We and others have recently shown that various ILC groups are present in both human and mouse uterus and, similar to ILCs in other tissues, uterine ILCs (uILCs) may also play roles in pregnancy (9, 10). For example, IFN-γ produced by uNK cells is the key factor required for uterine vascular adaptation during murine pregnancy (11), and various other ILC populations produce IFN-γ, including conventional (cNK) and tissue-resident NK cells (trNK), uterus and liver ILC1s, and gut NCR^+^ ILC3s (12). On the other hand, other cytokines produced by uILCs may affect pregnancy and reproduction, e.g., IL-5 produced by uILC2s and IL-17 and IL-22 produced by uILC3s (9, 10). Indeed, mice lacking lymphocytes, including uILCs, show impaired uterine arterial modifications (11) and fetal growth restriction (13). Defective uterine vascular remodeling and shallow trophoblast invasion are associated with great obstetrical syndromes, including preeclampsia and fetal growth restriction (14), and unbalanced cytokine production may lead to preterm labor (15).

Mice lacking the basic leucine zipper transcription factor NFIL3/E4BP4 display immune abnormalities (16), including severely reduced numbers of cNK cells (17) as well as reduced ILCs in several tissues (18–21). NFIL3 also regulates the emergence of common ILC precursors (22). However, the requirement for NFIL3 during development, homeostasis, and function of different ILCs is lineage and tissue specific. Indeed, *Nfil3^−/−^* mice retain trNK cells in uterus, liver, salivary glands, and skin, as well as thymically derived NK cells (23–25), and we have shown that they also have uILC3s (9). Moreover, the few residual cNK cells in *Nfil3^−/−^* mice exhibit normal effector functions in response to mouse cytomegalovirus infection (26). How the absence of NFIL3 impacts maternal responses to the endocrine and immunological changes occurring during gestation is unknown. We have begun to answer these questions by describing the changes in uILCs during pregnancy in *Nfil3^−/−^* mice mated with wild-type (WT) males. Using these mice, here we show that absence of maternal NFIL3 lead to a dramatic reduction of cNK cells and uILC2s during pregnancy. Despite the expansion of residual CD49^+^Eomes^+^ trNK cells and uILC3s and the presence of abundant CD49^+^Eomes^−^ uILC1s at midgestation, the uterine vasculature in *Nfil3^−/−^* females failed to complete the vascular remodeling process necessary to ensure ample and steady blood supply to the fetoplacental unit. This was associated with placental abnormalities and reduced fetal growth, although litter sizes were normal. The results demonstrate that NFIL3 regulates important functions for reproduction in mice, including key aspects of uILCs.

## Results and Discussion

### Curtailed Expansion of Nfil3^−/−^ uNK Cells in Response to Pregnancy

We asked if the residual uNK in virgin *Nfil3*^−/−^ females require NFIL3 to expand at midgestation. To do this, we quantified CD3^−^NK1.1^+^NKp46^+^ uNK numbers in both virgin and pregnant *Nfil3*^−/−^ females and compared them with numbers in WT females. Both *Nfil3*^−/−^ and WT females were mated with WT males to restrict NFIL3 deficiency to maternal tissues. Pregnant uteri were dissected into decidua – which is the transformed mucosa that forms in response to blastocyst implantation in mice – and the muscular outer layer, the myometrium, which includes a transient lymphoid structure that forms in rodents during pregnancy, the mesometrial lymphoid aggregate of pregnancy (MLAp). CD3^−^NK1.1^+^NKp46^+^ uNK expanded from 2.3 × 10^4^ cells in virgin to 13.7 × 10^4^ cells in pregnant WT mice (5.9-fold increase). The expansion in *Nfil3*^−/−^ mice was less pronounced, from 0.9 × 10^4^ cells in virgin to 2.4 × 10^4^ cells in pregnant mice (2.7-fold increase). Most of this expansion in both WT and *Nfil3*^−/−^ dams was due to accumulation of uNK cells in the myometrium (Table 1).

**Table 1:** **Numbers of uterine CD49a^+^ and CD49a^−^ NK cells in WT and *Nfil3*^−/^^−^ mice**.

	Spleen (×10^6^)	V. uterus (×10^4^)	Myometrium (×10^4^)	Decidua (×10^4^)
**WT mice**				
NK1.1^+^ NKp46^+^	1.613 ± 0.139	2.345 ± 0.358	11.9 ± 0.07	1.84 ± 0.031
cNK	1.515 ± 0.131	0.809 ± 0.206	2.215 ± 0.127	0.768 ± 0.070
CD49a^+^	0.098 ± 0.027	1.536 ± 0.293	9.581 ± 0.582	1.071 ± 0.229
***Nfil3*^−/−^ mice**				
NK1.1^+^ NKp46^+^	0.035 ± 0.023*	0.879 ± 0.044	1.932 ± 0.01***	0.459 ± 0.007**
cNK	0.019 ± 0.009*	0.057 ± 0.026*	0.030 ± 0.014***	0.012 ± 0.004***
CD49a^+^	0.008 ± 0.029*	0.816 ± 0.005	1.932 ± 0.763***	0.447 ± 0.053*

Leukocytes were enriched from virgin (v. uterus) and pregnant (gd9–10.5) uteri (myometrium separated from decidua) and stained for CD45, NK1.1, NKp46, CD3, DX5, and CD49a. Conventional NK cells (cNK) are identified as CD49a^−^DX5^+^. Based on percentages of positive cells, absolute cell numbers were calculated out of total live singlet CD45^+^ lymphocytes. Statistically significant differences between WT and *Nfil3*^−/−^ for each subset are indicated (*P < 0.05; **P < 0.01; ***P < 0.001, unpaired *t*-test, *n* = 2–5 independent experiments).

**Figure 1:**
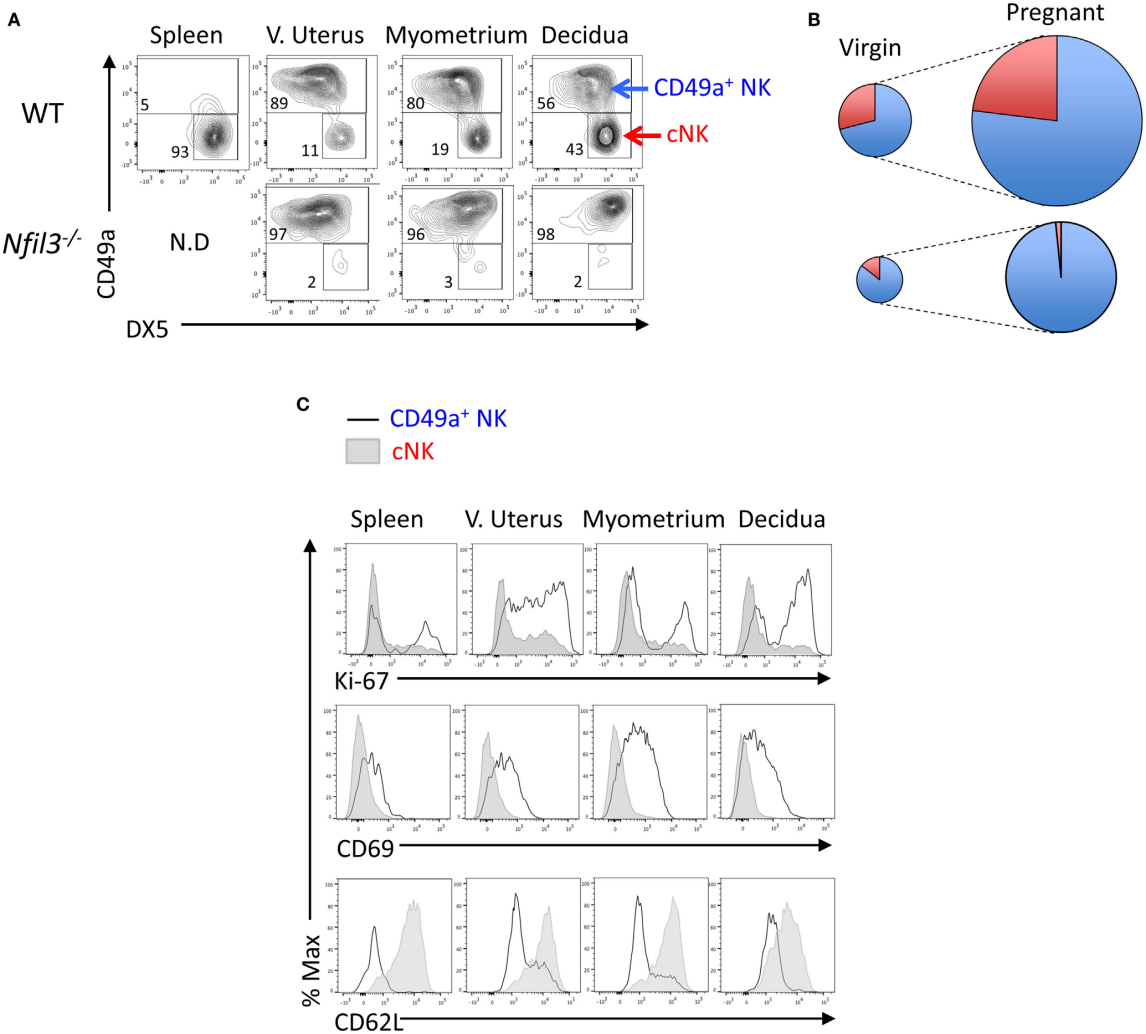
**Reduced expansion of uNK cells in *Nfil3^−/−^* dams**. **(A)** Representative flow cytometry plots showing separation of CD45^+^CD3^−^NK1.1^+^NKp46^+^ NK cells in CD49a^+^ uNK and CD49a^−^DX5^+^ (cNK) cells in the indicated tissues in WT and *Nfil3*^−/−^ females. **(B)** Visual representation of composition and expansion of CD49a^+^ uNK (blue) and CD49a^−^DX5^+^ cNK (red) in the uterus of virgin and pregnant WT and *Nfil3*^−/−^ mice. **(C)** Representative flow cytometry histograms of intracellular Ki-67, surface CD69 and CD62L in *ex vivo* CD49a^+^ uNK, and CD49a^−^DX5^+^ cNK in the indicated tissues of WT females. Data representative of two to three independent experiments, *n* = 4–20 mice per group.

Based on the expression of the two integrins CD49a (ITGA1, VLA-1) and DX5 (CD49b, ITGA-2, VLA-2), uterine CD3^−^NK1.1^+^NKp46^+^ cells can be divided in two subsets: CD49a^+^DX5^−^ and CD49a^−^DX5^+^ cells, which resemble similar subsets in liver trNK and cNK cells, respectively (25). Figure 1A shows a representative flow cytometry analysis, Figure 1B a diagrammatic representation, and Table 1 summarizes the quantification of the expansion of the two subsets during pregnancy. The expression of CD49a and DX5 has been described as mutually exclusive on the two subsets both in liver and uterus (25); however, we found that DX5 was expressed on most uterine CD49a^+^ cells. In *Nfil3*^−/−^ mice, the expression of DX5 on many CD49a^+^ cells increased at midgestation, albeit less than on cNK cells in WT mice (Figure 1A). NFIL3 may regulate expression of integrins (27), and we found that in *Nfil3*^−/−^ dams, expression of DX5 on some of the CD49a^+^ cells became even higher than that on cNK cells (Figure 1A).

*Ex vivo*, both CD49a^+^ cells and cNK cells stained positive for Ki-67, a marker strictly associated with cell proliferation (Figure 1C). Given the considerable expansion and the lower rate of proliferation in decidual cNK cells in WT mice (Figures 1A,C), it is conceivable that some of decidual cNK cells are cNK cells that came from the periphery. Another possible explanation is the *in situ* generation from progenitors, and both scenarios remain to be tested. Like the hepatic CD49a^+^ cells, also uterine CD49a^+^ cells were CD69^+^ and CD62L^−^ (Figure 1C).

### Phenotyping Uterine CD49a^+^ and CD49a^−^ Cells

**Figure 2:**
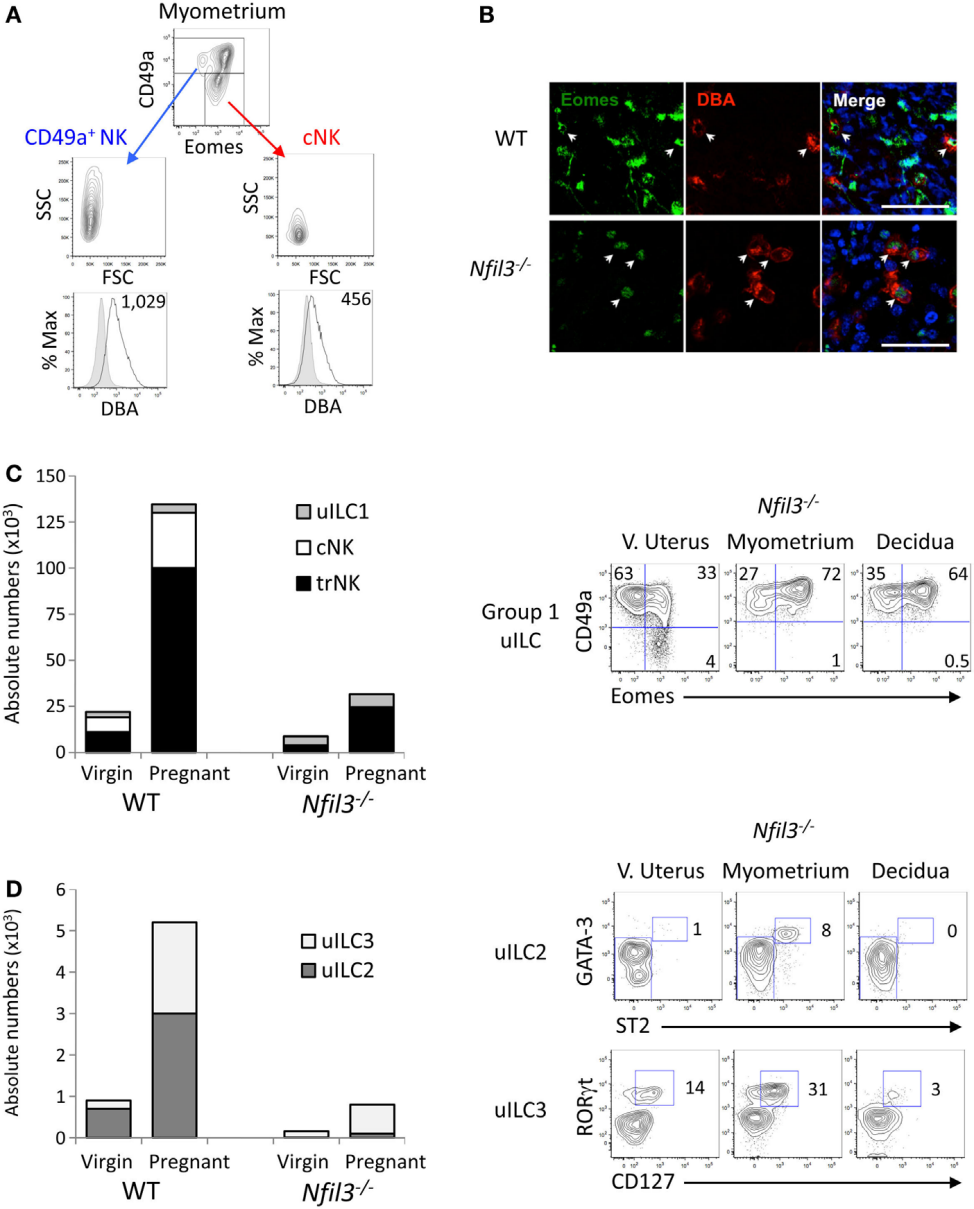
**Uterine ILCs in virgin and pregnant *Nfil3^−/−^* mice**. **(A)** Representative flow cytometry histograms of intracellular Eomes, granularity (SSC), size (FSC), and reactivity for *Dolichos biflorus* agglutinin (DBA) lectin (GMFI is indicated) in *ex vivo* CD49a^+^ NK and CD49a^−^ cNK in the indicated tissues of WT females. The negative control cell population for DBA reactivity is made up of CD3^+^ and CD19^+^ cells. **(B)** Representative immunofluorescence stainings of gd9.5 implantation sites (decidua) from WT and *Nfil3*^−/−^ pregnant females mated with WT males. Almost all DBA^+^ uNK (red) express intranuclear Eomes (green), as indicated by arrowheads. Scale bar = 50 μm. **(C)** Absolute numbers and composition in group 1 ILCs in the uterus from WT and *Nfil3*^−/−^ virgin and pregnant females (left graph). Representative flow cytometry plots of the changes in group 1 ILC composition from virgin to pregnant *Nfil3*^−/−^ mice (right panel). Gated on CD45^+^CD3^−^CD19^−^NK1.1^+^NKp46^+^ cells, uILC1 are defined as CD49a^+^Eomes^−^, uterine trNK cells as CD49a^+^Eomes^+^, and cNK cells as CD49a^−^Eomes^+^. Data representative of two to five independent experiments. **(D)** Absolute numbers and composition in uILC2s and uILC3s in the uterus from WT and *Nfil3*^−/−^ virgin and pregnant females (left graph). Representative flow cytometry plots of the changes in uILC2 and uILC3 composition from virgin to pregnant *Nfil3*^−/−^ mice (right panel). GATA-3^hi^ST2^+^ uILC2s are gated on CD45^+^CD3^−^CD19^−^CD11b^−^NK1.1^−^NKp46^−^CD90.2^+^ cells. RORγt^+^CD127^+^ uILC3s are gated on CD45^+^CD3^−^CD19^−^CD11b^−^NK1.1^−^CD90.2^+^ cells. Data representative of two to three independent experiments.

Expression of Eomes, among ILCs, defines NK lineages, whereas high granularity and staining with the lectin *Dolichos biflorus* agglutinin (DBA) in mice are features associated with uNK cells. We analyzed Eomes expression, granularity, and DBA reactivity on the two subsets of CD49a^+^ and CD49a^+^ uterine cells gated on CD45^+^CD3^−^CD19^−^NK1.1^+^NKp46^+^ cells. All CD49a^−^ cNK and most CD49a^+^ cells expressed Eomes in WT mice (Figure 2A), thus marking them as “bona fide” NK cells. The minority of the CD49a^+^ cells that did not express Eomes are Eomes^−^ uILC1s (9). uNK have also been known as “granulated” cells in several species (28). The granularity of CD49a^+^ cells was more pronounced than that of cNK cells (Figure 2A). Perhaps the most distinctive feature of mouse uNK cells is the positivity for DBA staining (29), although more recently, it has become apparent that some uNK cells are not reactive for DBA (30, 31), may be the largest producer of IFN-γ (32) and, if inhibited, contribute to insufficient uterine adaptations to pregnancy (13). DBA staining mostly colocalized with Eomes staining in tissue sections of both WT and *Nfil3*^−/−^ dams (Figure 2B). Although both CD49a^+^ cells and cNK cells stained positive for DBA, CD49a^+^ cells showed a brighter staining than cNK cells (Figure 2A), suggesting that these cells may relate more closely to what traditionally has been referred to as a major subset of uNK cells. In line with this, CD49a^+^ cells produced less IFN-γ than cNK cells after stimulation with PMA and ionomycin or with IL-12 and IL-15 (not shown).

### Expansion of Group 1 Uterine ILC1 Subsets in Pregnancy

The data here and those in the accompanying manuscript (33) corroborate our recently published data, which show that Eomes expression defines two populations of group 1 ILCs within the CD49a^+^ subsets, which, along with cNK cells make up the three subsets of group 1 uILC1, defined collectively as CD45^+^CD3^−^CD19^−^NK1.1^+^NKp46^+^ cells (9). The three subsets are CD49a^+^Eomes^+^ trNKs, CD49a^+^Eomes^−^ ILC1s, and CD49a^−^DX5^+^ cNK cells (9). We next set out to define how pregnancy and the lack of NFIL3 affect the changes in these three subsets of group 1 ILC1s. Eomes^+^ trNK cells expanded about sevenfold in *Nfil3*^−/−^ mice (Figure 2C). This expansion was nearly as extensive as in pregnant WT mice; however, trNK cells were still over fivefold less numerous in *Nfil3*^−/−^ dams than in WT dams, presumably because they were already decreased in virgin mice. Eomes^−^ uILC1s did not seem to expand in *Nfil3*^−/−^ dams and expanded very little in pregnant WT dams too. CD49^−^DX5^+^ cNK cells did not expand at all in *Nfil3*^−/−^ dams, whereas they expanded 3.7-fold in WT dams (Figures 1A and 2C; Table 1). These results show that the population of uterine group 1 ILC that expand the most during pregnancy in WT mice is trNK cells, with some expansion of cNK cells and little or no expansion of Eomes^−^ uILC1 cells (Figure 2C; Table 1). NFIL3 is required for development and expansion of uterine cNK cells and for development of trNK cells, but is dispensable for the expansion of trNK cells and the development of Eomes^−^ uILC1s.

### Expansion of Uterine ILC2 and ILC3 in Pregnancy

NFIL3 is important also for the development of other ILCs. *Nfil3*^−/−^ mice are severely deficient in ILCs in several tissues, such as the intestine or the lungs (19–21). Recently, we have shown that only uILC2s strictly require NFIL3 to develop in the uterus of virgin mice (9). Indeed, uILC2s were not detectable in virgin *Nfil3*^−/−^ mice and barely detectable in pregnant *Nfil3*^−/−^ mice (Figure 2D), and uILC3s were present in virgin *Nfil3*^−/−^ mice but they did not expand as in WT mice during pregnancy (Figure 2C).

It will be interesting to study the potential role of uILC2s and uILC3s during murine pregnancy, for example, in MLAp formation. Moreover, ILC3s are the most abundant non-NK ILCs in the human uterus (9, 10), and they may play roles in human pregnancy.

Altogether, the data show that NFIL3 may serve different roles in different uILC lineages. Homeostatic development of trNK in virgin mice, but not expansion in response to pregnancy, requires NFIL3, whereas the opposite seems to be true for uILC3s. On the other hand, cNK and uILC2s require NFIL3 for both development and expansion.

### Maternal, Placental, and Fetal Abnormalities in Nfil3^−/−^ Dams

Our data show that *Nfil3*^−/−^ mice exhibit defects in the development and/or the expansion of uterine cNK cells and other uILCs during pregnancy. The residual trNK cells, as well as the Eomes^−^ uILC1s and the uILC3s found in *Nfil3*^−/−^ dams, may however sustain the uterine adaptations necessary for placentation and fetal growth. In order to test this, we compared uterine arterial changes, litter sizes, and placental and fetal growth in WT and *Nfil3*^−/−^ dams mated with syngeneic WT mice. In contrast to what was found in allogeneic pregnancies (34), we found normal litter sizes. Therefore, the lack of NFIL3 does not impact on the number of live births in our crosses. Previous work with mice that do not develop uNK cells or have hypofunctional uNK cells showed that these mice too have normal litter sizes. However, analysis of uterine tissues and fetoplacental growth revealed clinically relevant abnormalities in these mice (11, 13).

**Figure 3:**
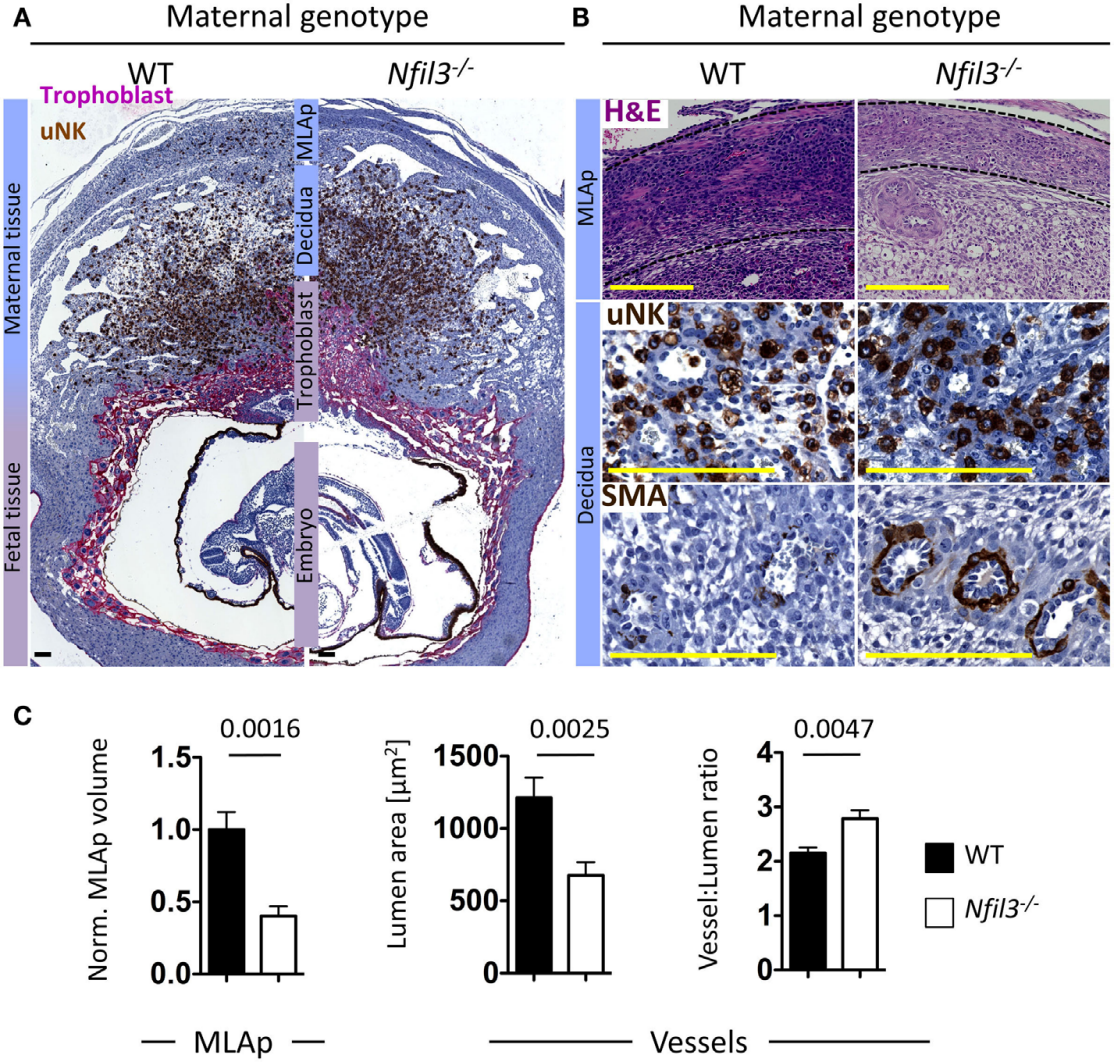
**Abnormalities in uterine tissues of *Nfil3^−/−^* dams**. **(A)** Representative immunohistological staining of gd9.5 implantation sites from WT and *Nfil3*^−/−^ pregnant females mated with WT males. DBA^+^ uNK cells (brown) within the mesometrial lymphoid aggregate of pregnancy (MLAp) and the decidua and in proximity of the invading trophoblast, which is stained with cytokeratin (purple). Bar = 500 μm. **(B)** H&E and immunohistological staining of MLAp and decidua at higher magnification showing MLAp area, distribution of uNK around vessels, and presence of residual smooth muscle actin (SMA) within the vascular wall. Bar = 500 μm. Indicated is the maternal geotype. All females were mated with WT males. **(C)** Stereological quantification of MLAp (left), vascular lumen area (middle), and relative wall thickness (right). Means ± SEM of data representative of 4–6 litters per genotype. Statistical differences were calculated with an unpaired Student’s *t*-test.

**Figure 4:**
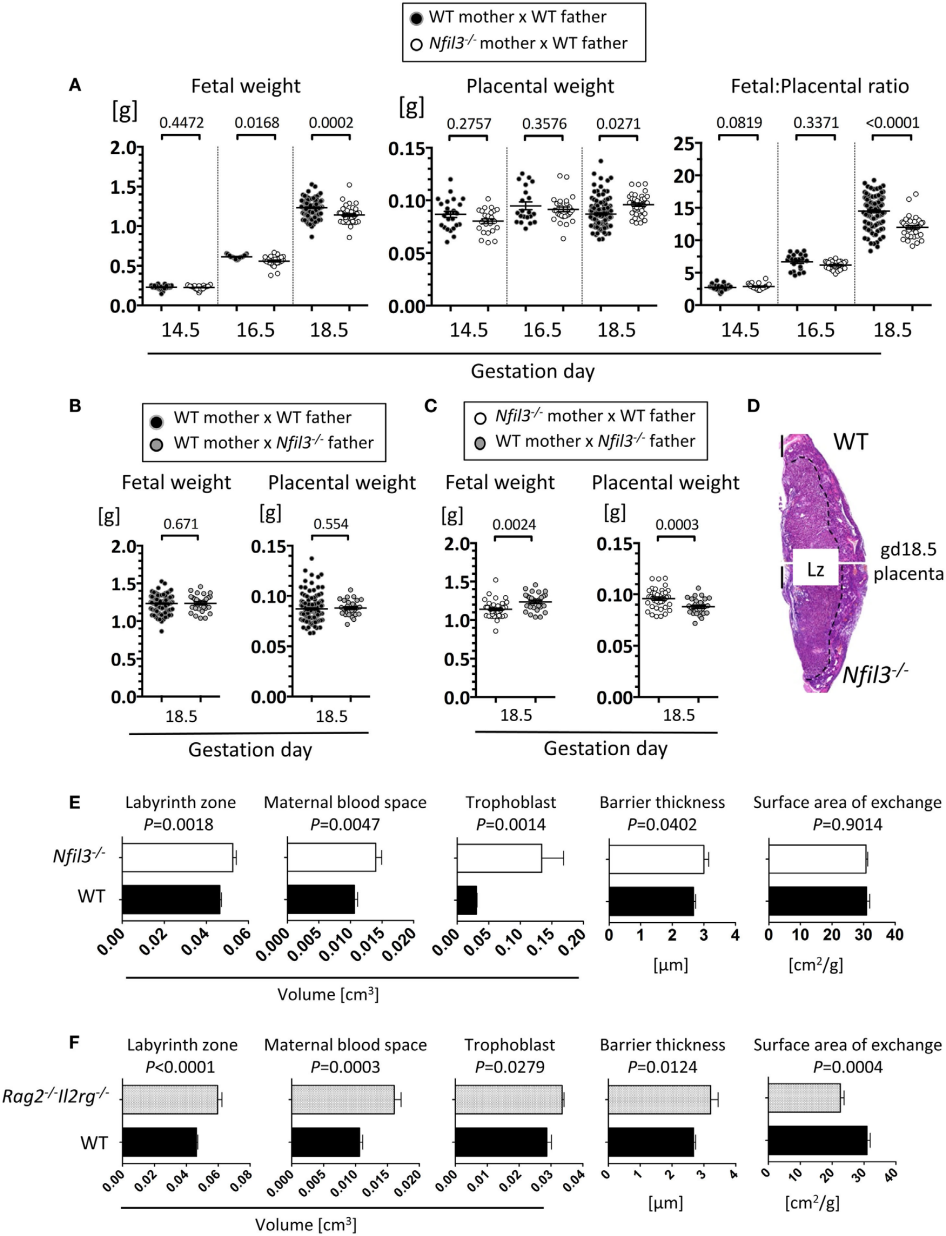
**Fetal and placental abnormalities in *Nfil3^−/−^* dams**. **(A)** Fetal weight, placental weight, and ratio of fetal/placental weight of conceptuses from either WT or *Nfil3*^−/−^ females mated with WT males. Means ± SEM of data representative of 3–18 litters per maternal genotype per gestational age. **(B)** Fetal and placental weights of conceptuses from WT females mated either with WT or *Nfil3*^−/−^ males. Means ± SEM of data representative of 4–5 litters per maternal genotype. **(C)** Fetal and placental weight of heterozygous offspring from either *Nfil3*^−/−^ dams **(A)** or *Nfil3*^−/−^ fathers **(B)**. Means ± SEM of data representative of 4–5 litters per cross. **(A–C)** Statistical differences were calculated using mixed effect modeling taking into account the clustering of pups from the same litter. **(D)** Representative H&E staining of gd18.5 placenta from WT and *Nfil3*^−/−^ dams. The discontinuous line demarcates the labyrinth zone (Lz). **(E,F)** Stereological evaluation of the volume of labyrinth zone, maternal blood space, trophoblast, barrier thickness, and surface area of exchange in placentas from *Nfil3*^−/−^ dams **(E)** and in placentas from lymphocyte deficient *Rag2*^−/−^*Il2rg*^−/−^ dams **(F)**, both compared to those from WT dams. Data representative of 5–11 placentas per genotype for Lz volume and 4–9 placentas for Lz structure (MBS, trophoblast, BT, and SA). Statistical differences were calculated with an unpaired Student’s *t*-test.

To assess the role of residual ILCs in *Nfil3*^−/−^ mice during pregnancy, we used stereology and immunohistochemistry to analyze morphological aspects of implantation sites in *Nfil3*^−/−^ females mated with WT males. DBA^+^ uNK cells were readily detected in uterine sections of both WT and *Nfil3*^−/−^ mice, including in the decidua and in the MLAp (Figures 3A,B). The MLAp is reduced in size in NK-deficient mice (11) or in mice engineered to have hypofunctional uNK cells (13), and we found here that it was also the case in *Nfil3*^−/−^ dams (Figures 3A,B). Vascular walls were thicker with incomplete loss of smooth muscle actin (SMA) in *Nfil3*^−/−^ dams (Figures 3B,C), a phenotype typical of NK-deficient mice (11, 13). Impaired arterial remodeling in uNK-deficient mice results in placental abnormalities and fetal growth restriction. *Nfil3*^−/−^ dams and their fetuses displayed phenotypes similar to those of NK-deficient mice (Figure 4A). Conversely, WT females mated with *Nfil3*^−/−^ males had normal-sized fetuses and placentas (Figure 4B). The comparison of *Nfil3*^−^*^*/*^*^+^ with *Nfil3*^+^*^*/*^*^−^ conceptuses showed that the reduction in fetal weight and the increase in placental weight is a phenotype of maternal origin (Figure 4C).

The labyrinth zone of mouse placenta is analogous to the human placental villi, where nutrient and gaseous exchange take place. The volume of the labyrinth zone was larger in placentas of *Nfil3*^−/−^ dams (Figures 4D,E). Trophoblast and maternal blood spaces, as well as the thickness of the barrier separating the fetal and maternal blood spaces, were increased in placentas of *Nfil3*^−/−^ dams (Figure 4E). These abnormalities, with the exception of the surface area of placental exchange, mirror those in NK-deficient *Rag2*^−/−^*Il2rg*^−/−^ dams (Figure 4F) and are consistent with altered placental hemodynamics secondary to deficient spiral artery remodeling (35, 36). The reason and the significance of the divergent phenotype of the surface area of placental exchange in *Nfil3*^−/−^ and *Rag2*^−/−^*Il2rg*^−/−^ dams are unclear; however, it may reflect the difference in cellularity in the two strains, with residual ILCs and other lymphocytes in *Nfil3*^−/−^ dams and total lack of all lymphoid cells in *Rag2*^−/−^*Il2rg*^−/−^ dams.

In a mouse model of allogeneic pregnancy, Fu et al. found increased Th17 cells in the decidua of *Nfil3*^−/−^ dams, which was associated with fetal demise, and they suggested that the absence of uNK cells in *Nfil3*^−/−^ dams might have caused Th17 expansion and breakdown of tolerance (34). We find here that some NK are instead present in *Nfil3*^−/−^ dams at midgestation, alongside Eomes^−^ uILC1s, and uILC3s. In conclusion, our results show that the residual uILCs in *Nfil3*^−/−^ dams are not sufficient to mediate normal uterine adaptations to pregnancy. Placentation and fetal growth is compromised in the absence of NFIL3, which emerges as an essential mediator of non-redundant functions for reproduction in mice.

## Materials and Methods

### Mice

C57BL/6J mice were purchased from Charles River UK and were used as WT mice in all experiments. *Nfil3*^−/−^ and *Rag2*^−/−^*IL2rg*^−/−^ mice were in C57BL/6J background as previously described (17, 37). All mating experiments used virgin females mated either with C57BL/6J males or with *Nfil3*^−/−^ males as indicated. Gestation day (gd) 0.5 was counted at noon of day of the appearance of a copulation plug. Virgin and pregnant females were age matched (7–12 weeks). All procedures were conducted in accordance with the University of Cambridge Animal Welfare and Ethical Review Body and United Kingdom Home Office Regulations.

### Cell Preparation

Ovaries, mesometrium, and cervix were removed from all uteri in ice-cold PBS without Ca^2+^/Mg^2+^. Virgin uteri were entirely minced with scissors. For gd9.5 and gd10.5 mid-gestated uteri, myometrium tissue (including MLAp) were first dissected from the decidua and pooled. The decidua basalis (containing placenta) tissues were also separately minced and pooled. Embryos, yolk sacs, and decidua parietalis were discarded. Minced tissues were first softened in the predigestion solution, 1× HBSS (PAA) containing 5 mM EDTA (Sigma), 15 mM HEPES solution (Life Technologies), and 10% FCS (Life Technologies) under moderate rotation (220 rpm) for 2 × 15 min at 37°C. Then, the cell suspension was filtrated through a 100-μm cell strainer and the minced tissues were rinsed from remaining EDTA with 1× PBS. This flow-through fraction containing intraepithelial lymphocytes is stored on ice by the time the minced tissue undergoes enzymatic digestion. The latter are dissociated in RPMI 1640 containing 2% FCS, 0.1 WU/ml Liberase DH, and 30 μg/ml DNase (Roche) for 30 min at 37°C under gentle agitation. Digested tissues were fully dissociated with a syringe plunger against a 100-μm cell strainer. Pre-digest and post-digest flow-through solutions were pooled and washed in Ca^2+^/Mg^2+^-free PBS containing 5 mM EDTA for enzyme inactivation. The cell suspension was overlayed on a 80/40% Percoll (GE Healthcare Life Sciences) gradient and the leukocyte fraction was collected at the interface. Cells from spleens and livers (from virgin mice) were similarly prepared except from the EDTA pre-digest step [adapted from Ref. (38, 39)].

### Flow Cytometry

Conjugated Abs anti-mouse CD45 (clone 30-F11), CD3ϵ (500A2 or 17A2), CD19 (6D5), NK1.1 (PK136), NKp46 (29A1.4), CD90.2 (30-H12), ST2 (RMST2-2), CD49b (DX5), NK1.1 (PK136), NKp46 (29A1.4), CD62L (MEL-14), CD127 (A7R34), RORγt (Q31-378), anti-human/mouse CD11b (M1/70), Eomes (Dan11mag) and CD16/32-Fc blocking (93), anti-rat/mouse CD49a (Ha31/8), anti-human/mouse GATA-3 (TWAJ and 16E10A23), and Ki-67 (B56) were purchased from Biolegend, eBioscience, BD Biosciences, or R&D Systems. Transcription factors and Ki-67 were stained using the FoxP3 staining buffer set (eBioscience) according to the manufacturer’s instructions. Fixable viability dyes eFluor 780 and eFluor 506 (eBioscience) was used to exclude dead cells. Cells were stained with DBA (Vector Labs) after the fixation/permeabilization step. Samples were acquired on a LSR Fortessa (BD Biosciences) using FACS DiVa and analyzed using FlowJo (Tree Star).

### Immunohistochemistry

Total pregnant uteri were fixed 6 h at room temperature (RT) in formalin and embedded in paraffin. Sections were serially cut at 7 μm and stained with hematoxylin and eosin (H&E) at 49 μm intervals using standard methods. For chromogenic IHC cytokeratin/DBA double staining, proteinase-K mediated antigen retrieval was performed, and sections were incubated with polyclonal rabbit anti-cytokeratin (1:1000, DAKO, Z0633) at 4°C, followed with biotinylated universal pan-specific antibody (1:500, Vector lab, BA-1300) for 1 h at RT and labeled using VECTASTAIN ABC-AP KIT (Vector lab, AK-5000) and SIGMA *Fast* (Sigma-Aldrich, F4523). After colorimetric detection, sections were blocked using Avidin/Biotin Kit (Vector lab, SP-2001) and incubated with biotinylated DBA-lectin (6.6 μg/ml) for 30 min at RT, labeled using ABC kit (Vector lab, PK6100) and DAB, Diaminobenzidine (Sigma-Aldrich, D4168). For SMA, SMA single staining, paraffin sections were subjected to citrate buffer heat-induced epitope retrieval (HIER) for 3 min, blocked using Mouse On Mouse (MOM) kit (Vector lab, MKB-2213), and incubated with mouse anti-human SMA (1:100, DAKO, M0851) for 30 min at RT, followed by MOM biotinylated anti-mouse IgG reagent (Vector lab, MKB-2213), labeled using ABC kit (Vector lab, PK6100) and DAB (Sigma-Aldrich, D4168) and counterstained with hematoxylin. All immunohistochemical stainings included isotype controls. For dual immunofluorescence staining of DBA and Eomes, 7 μm thick cryosections of uterine tissue at gd9.5 were fixed in 100% acetone at 4°C for 20 min. Sections were incubated with AF488-conjugated rat anti-mouse Eomes (eBioscience, Dan11mag, 1:50) for 60 min at RT and sequentially with FITC-conjugated donkey anti-rat IgG (Jackson Immunoresearch, 712-095-153, 1:150), AF488-conjugated rabbit anti-fluorescein/Oregon Green (Life Technologies, A-11090, 1:200), and AF488-conjugated donkey anti-rabbit IgG (Life Technologies, A-21206, 1:100) for 30 min each at RT. Sections were incubated with 6.6 μg/ml biotinylated DBA and labeled using AF555-conjugated streptavidin (Life Technologies, S-32355, 1:500), each for 30 min at RT. Sections were counterstained with DAPI. Images were acquired using a Leica SP5 confocal microscope and analyzed using ImageJ software to adjust brightness and contrast.

### Stereology Analysis of MLAp Volume and Decidual Arteries

Tissue volumes were quantified from serial sections using the Cavalieri method (40). Briefly, MLAp cross-sectional areas were calculated on serial sections and volumes were calculated by accounting for the spacing between the sections as previously described (41). Arterial remodeling was evaluated in the central decidua basalis on sagittal sections and veins localized at the periphery were excluded. Three to five implantation sites from randomly chosen uterine horn were analyzed per litter. In every implantation site, three to five largest vessels were measured in triplicate (three sections with distance of 49 μm apart). Lumen area and vessel-to-lumen ratios were quantitated by using outlines tools from NDP viewer (Hamamatsu) and Aperio ePathology (Leica Biosystems) softwares. SMA IHC staining was used for further qualitative assessment of arterial remodeling (41).

### Stereology Analysis of Placentas

Midsagittal cross-sections of placental tissue (gd18.5) were stained with H&E using standard protocols. From each of nine WT and five *Nfil3*^−/−^ mice, the placenta closest in weight to the litter mean was selected for stereology analysis. The fractional volumes of the labyrinth zone (Lz) were assessed by point counting (10× objective lens, the newCAST Computer Assisted Stereological Toolbox system Visopharm, Hoersholm, Denmark) with systematic random sampling (42). Assuming a density of 1 mg mm^−3^, fractional volumes were converted to absolute volumes in cubic millimeter by multiplying by total placental volume (43). The other placental halves were fixed in glutaraldehyde and embedded in Spurr’s epoxy resin and a single mid-line section taken (1 μm thickness) then stained with toluidine blue. Volumes of maternal blood space and trophoblast in the Lz, surface area, and thickness of the barrier were calculated at 100× magnification using stereological methods that were described previously (42).

### Statistics

Data were analyzed using paired or unpaired parametric Student’s *t*-test or non-parametric Mann–Whitney test. Fetal and placental weights were analyzed using a mixed model approach to test the effect of maternal genotype and account for both gestational age and clustering of observations by litter (44). *P* < 0.05 was taken as statistically significant for all tests. Analyses were performed using GraphPad Prism and IBM SPSS.

## Author Contributions

HB, AM, and FC conceived the project. SB, J-MD, AS-P, LG, JK, EB, HY, SJ, LV, DH, and KP performed experiments. SB, J-MD, AS-P, LG, JK, AF, GB, and FC designed experiments and analyzed data. SB, J-MD, and FC wrote the manuscript.

## Conflict of Interest Statement

The authors declare that the research was conducted in the absence of any commercial or financial relationships that could be construed as a potential conflict of interest.
